# On the Good Effect of Æther and Tinct. Opii, in Cramp of the Stomach

**Published:** 1800-11

**Authors:** Sam. Thomas

**Affiliations:** Surgeon. Wakefield, Yorkshire


### *On the good Effect of Æther and Tinct. Opii, in Cramp of the Stomach.*

ON the 2d of September, a lady was attacked (though in previous health) with violent pain of the back while ftooping, which immediately extended from the fpine to the fternum, and fixed upon the region of the ftomach, and was fo violent as to caufe clammy fweats, a moift, pale, cadaverous look, juft as if fhe was dying; pulfe hardly perceptible; and, to exprefs her own feelings, faid fhe thought fhe was dying. As foon as poffible I got her fome æther and tinct. opii, which eafed her a little, fo that fhe was able to get up ftairs to bed, and applied warm flannel, &c. to the ftomach; but the pain became more fevere, attended with violent fhivering, which continued for the fpace of twenty minutes; clammy fweats and palenefs; pulfe hardly perceptible, and the extremities cold. Being now very much alarmed, Dr. Crowther, a phyfician of eminence in this town, was fent for; he came immediately, and requefted me to continue to give her æther ʒj. tinct. opii gt. xv.; to rub the back with æther, &c. and for her to take it every hour, if the pain continued violent; to apply hot cloths, wrung out of water, to her feet. Thefe remedies were perfifted in for an hour or two, and the æther, &c. gave almoft inftant relief. The ftomach was fo weak that fhe was not able to bear any thing ftronger than a little faloop, in which there was put a fmall quantity of brandy. In the evening, being coftive, had a clyfter, and the pain in the ftomach better; but could not bear up in an erect pofture of body, as, if fhe did, the pain returned with violence; but always found relief from a dofe of the æther.

On the 3d fhe was better, except the pains coming on at flight intervals; but could not yet bear up in an erect pofture of body. Continued the æther and tinct. opii ter die, et enema nocte.

4th. Still continues better, but the ftomach fo weak as not to take broth; ftill finds herfelf eafy when laid down; in the erect pofture very uneafy. Æther, &c. continued.

As it would be ufelefs to give every day's account, which would be only trefpaffing on your time, I have only to obferve that fhe has experienced flighter attacks, which have not been fo violent as the firft, but that fhe has always found immediate relief from æther, and which fhe thinks was evidently one means of faving her life.

The above cafe certainly fhews us the good effects of æther; for for if the debilitating syſtem had been made uſe of, probably the lady would not at this time have been living.

Having obſerved in your Journal the good effect of ol. terebinth. in burns and ſcalds; a near relation of mine happened to ſcald herſelf with ſome boiling broth, which cauſed intenſe heat and inflammation, but on pouring ol. terebinth. upon the part, for the ſpace of a quarter of an hour, it gave her eaſe, nor was any ill effects produced by the ſcald.

*Wakefield, Yorkſhire,* SAM. THOMAS, Surgeon.

